# Bioactive Compounds and Antioxidant Efficacy of Djulis (*Chenopodium formosanum*) Leaves: Implications for Sustainable Cosmeceutical Development

**DOI:** 10.3390/antiox14020202

**Published:** 2025-02-10

**Authors:** Chien-Shan Chiu, Yung-Jia Chan, Yan-Zhen Wu, Wen-Chien Lu, Po-Yuan Chiang, Po-Hsien Li

**Affiliations:** 1 Department of Dermatology, Taichung Veterans General Hospital, Taichung 40705, Taiwan; cschiu@nchu.edu.tw; 2 Department of Post-Baccalaureate Medicine, College of Medicine, National Chung Hsing University, Taichung 40227, Taiwan; 3 College of Biotechnology and Bioresources, Da-Yeh University, Changhua 51591, Taiwan; d0867601@cloud.dyu.edu.tw; 4 Department of Food and Nutrition, Providence University, Taichung 43301, Taiwan; 5 Department of Food and Beverage Management, Chung-Jen Junior College of Nursing, Health Sciences and Management, Chia-Yi City 60077, Taiwan; m104046@cjc.edu.tw; 6 Department of Food Science and Biotechnology, National Chung Hsing University, Taichung 40227, Taiwan

**Keywords:** *Chenopodium formosanum*, djulis leaves, antioxidant, cosmeceuticals

## Abstract

*Chenopodium formosanum* (djulis), well known for its vivid color variation during diverse senescence stages, has attracted attention for its perceived health benefits and antioxidant capacity. Djulis leaves, often discarded as biowaste after harvesting, were evaluated for their potential as a source of antioxidant compounds. The current study analyzes the physicochemical and antioxidant activities of red, green, and yellow djulis leaf extracts across various senescence stages to probe their prospective utility in cosmeceuticals. Various plant compounds including total carbohydrates, phenolics and flavonoids, chlorophyll a and b, and betaxanthins and betacyanins were measured using spectrophotometric techniques. Antioxidant potential was assessed using DPPH, FRAP, CUPRAC, TEAC, and DMPD assays. Green djulis leaves displayed elevated total carbohydrate and chlorophyll levels, whereas red djulis leaves exhibited heightened phenolic, flavonoid, betaxanthin, and betacyanin content, indicating its suitability for cosmeceutical applications. Antioxidant evaluations revealed substantial disparities among the extracts, with red djulis leaf extract demonstrating superior antioxidant activity in most assays. These findings revealed the distinct antioxidant profiles of djulis leaf extracts influenced by color and senescence stage. These findings advance our understanding of the bioactive attributes of djulis leaves and their potential for incorporation into functional products.

## 1. Introduction

*Chenopodium formosanum*, commonly known as djulis, has a significant place in Taiwanese culture and traditional medicine because of its rich nutritional content and purported health benefits [1]. Djulis, a member of the Amaranthaceae family, is indigenous to Taiwan, where it has been cultivated and consumed for centuries [2]. Beyond its aesthetic appeal, djulis contains a treasure trove of bioactive compounds with potential health-promoting properties [3,4]. Among these, antioxidants have garnered considerable attention because of their ability to neutralize harmful free radicals and mitigate oxidative stress, a process implicated in various chronic diseases and aging [5]. The antioxidant capacity of djulis is attributed to their phenolic compounds, vitamins, and minerals, which act synergistically to scavenge free radicals and modulate oxidative pathways within the body [6]. Previous studies have investigated the effect of bioactive compounds on vasodilation, angiotensin-converting enzyme activity (ACE), and hypertension in vitro and in vivo, demonstrating that aqueous extracts of djulis displayed an obvious inhibitory effect on ROS generation, peroxynitrite levels, and ACE activity [7].

The seeds of djulis have been extensively utilized in food manufacturing and pharmaceutical research [8]; however, their leaves have long been underappreciated. In addition to its seeds, djulis is characterized by its strikingly colorful leaves, transitioning from shades of green to red and yellow as they progress through various senescence stages [9]. In the early stages of growth, djulis leaves are predominantly green due to the high concentration of chlorophyll [10]. As the plant matures or under certain environmental conditions, the amount of chlorophyll may decrease, and other pigments like betacyanins become more dominant [11]. Betacyanins are part of the betalain pigment family and are responsible for the red-to-purple colors in djulis [12]. These pigments absorb green light and reflect red and violet light, giving the leaves a reddish hue. Betacyanins also have antioxidant properties, which help protect the plant from environmental stress, such as UV radiation and oxidative stress [13]. The yellow color in djulis leaves is due to the presence of pigments like betaxanthins [14]. Betaxanthins, another group of betalain pigments, are responsible for the yellow-to-orange colors [14]. As the plant ages, chlorophyll may break down, causing the yellow pigments to become more prominent [15]. However, the leaves of djulis have often been overlooked and considered waste materials even though djulis leaves are allowed to be used as food ingredients by the Taiwan Food and Drug Administration (TFDA). Nevertheless, the distinct features of djulis leaves have captured the attention of researchers and enthusiasts alike, prompting investigations into the underlying factors influencing their coloration and nutritional profile. Recent studies have highlighted the potential value of djulis leaves as a rich source of bioactive compounds, including antioxidants and phytochemicals [16]. There is an increasing interest in utilizing agricultural by-products and waste materials for sustainable product development [17].

In recent years, there has been growing interest in utilizing natural plant extracts as antioxidants for developing cosmeceutical products in the cosmetic and skincare industries [18]. Cosmeceuticals, a hybrid category of cosmetics and pharmaceuticals, offer functional benefits beyond traditional skincare by targeting specific skin concerns while providing nourishment and protection. Related studies using unhulled red djulis extracts to treat human fibroblasts (CCD-966SK cells) demonstrated that unhulled red djulis extracts effectively increased the repairing ability of skin cells and amplified skin-barrier-related and antioxidant-related gene expression [19]. Furthermore, unhulled red djulis extracts increased collagen-related gene expression and inhibited AGEs and melanin formation [19]. Additionally, unhulled djulis was extracted using water at a ratio of 1:10 (*w*/*v*), which decreased UV-induced intracellular ROS generation and triggered the antioxidant defense system by activating the nuclear factor erythroid 2-related factor 2 (Nrf2)/heme oxygenase-1 (HO-1) signaling pathway in human skin fibroblasts (Hs68 cells) [20]. Djulis extracts, with their potent antioxidant activity, are promising natural ingredients for cosmeceutical formulations aimed at addressing oxidative-stress-induced skin damage, premature aging, and other dermatological conditions. Nevertheless, the use of djulis leaf extract in cosmeceutical applications is still not well researched.

Despite the growing interest in djulis leaves as a potential source of antioxidants for cosmeceutical applications, comprehensive studies examining the antioxidant profiles of leaves of different colors at various senescence stages remain limited. Understanding how color variation and senescence influence the antioxidant composition of djulis leaves is essential for optimizing their efficacy and safety in cosmeceutical formulations. Elucidating the bioactive constituents responsible for the observed antioxidant activity will facilitate the development of standardized extracts of consistent quality and potency.

Based on the factors mentioned above, this study aimed to investigate the antioxidant activities of red, green, and yellow djulis leaf extracts, taking into account their distinct senescence stages. Through a comprehensive analysis of their antioxidant profiles, this study provides valuable insights into the potential applications of djulis leaves in cosmeceuticals and other functional products. By harnessing the natural antioxidant properties of djulis leaves, this study seeks to contribute to the development of innovative skincare formulations that promote skin health and vitality, reflecting the rich botanical heritage of Taiwan and its indigenous plant species. By using djulis leaves as a material, this study may contribute to the valorization of agricultural waste and the development of value-added products with potential benefits for both human health and environmental sustainability.

## 2. Materials and Methods

### 2.1. Materials and Sample Preparation

**Figure 1:**
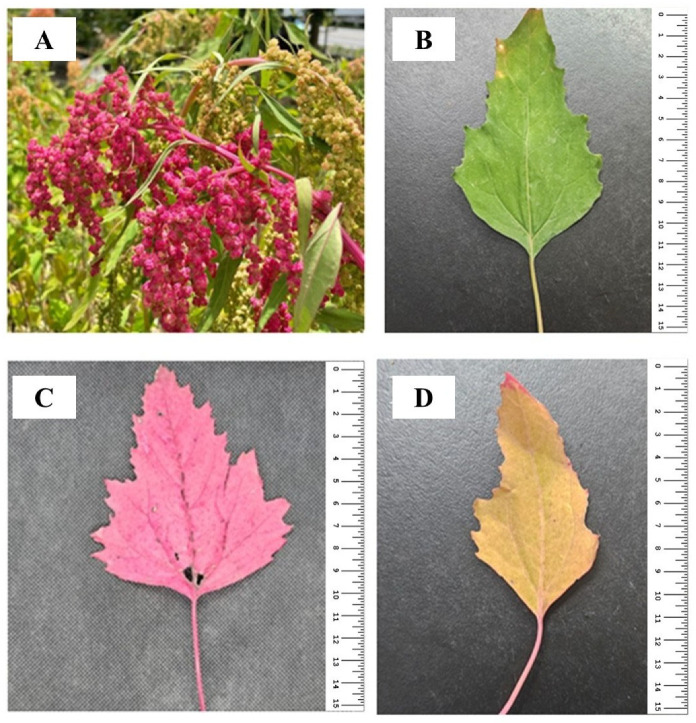
(**A**) Djulis (*Chenopodium formosanum*). (**B**) Green djulis leaf. (**C**) Red djulis leaf. (**D**) Yellow djulis leaf.

Djulis (*Chenopodium formosanum* Koidz), cultivated in Yunlin County (Taiwan), was supplied by Hearty Farm (Yunlin, Taiwan) (Figure 1A). The leaves of djulis were separated by color (Figure 1B–D) before being oven-dried at a temperature of 40 °C, powdered using a blender (model CB-5A, Chuang Pao Special Precision Industry Co., Ltd., Kaohsiung City, Taiwan), and passed through a 0.5 mm mesh. For the extraction, 10 g of the powder was mixed with 100 mL of distilled water and stirred (150 rpm) at 4 °C overnight. After 24 h, the mixture was filtered, and the filtrate was frozen and lyophilized in a freeze dryer (Uniss FDM-20 Freeze Dryer, Xinbei City, Taiwan). The chemicals used in this study, such as sulfuric acid, Folin-Ciocalteu reagent, gallic acid, ferric acid, sodium hydroxide, and dimethyl sulfoxide (DMSO), were of analytical grade (purity > 99%) and purchased from Sigma-Aldrich (St. Louis, MO, USA).

### 2.2. Physicochemical Properties

#### 2.2.1. Total Carbohydrate

The freeze-dried sample powder (4.2 mg) was dissolved in distilled water to a final volume of 25 mL. After adding 1 mL of the sample solution to 1 mL of 5% phenol solution, 5 mL of 98% concentrated sulfuric acid was quickly added. After thorough mixing, the solution was left at room temperature for 15 min and the absorbance was measured at 490 nm using a spectrophotometer (SH-U830, Shishin Technologies Co., Ltd., Taipei City, Taiwan). A standard curve was created using D-glucose concentrations ranging from 0.009 to 0.100 mg glucose/mL (Figure S1A) while the result was performed in mg glucose/g to determine the total carbohydrate content (mg glucose/g DW) of the samples.

#### 2.2.2. Total Phenolic Content (TPC)

A total of 5.6 mg of freeze-dried sample powder was dissolved in distilled water to a final volume 5 mL. Next, 1 mL of the sample solution was mixed with 1 mL of 0.5 Folin-Ciocalteu reagent and 2 mL of 20% sodium carbonate (Na_2_CO_3_). After mixing thoroughly, the absorbance was measured at 750 nm. The TPC (mg gallic acid/g) of the sample was determined using a standard curve prepared with gallic acid (0.001–0.04 mg gallic acid/mL) (Figure S1B). In contrast, the results in the samples will be expressed in mg gallic acid/g of the lyophilized sample.

#### 2.2.3. Total Flavonoid Content (TFC)

Freeze-dried sample powder (8.2 mg) was dissolved in distilled water to a final volume of 5 mL. In total, 1 mL of the sample solution was mixed with 0.3 mL of 5% sodium nitrite (NaNO_2_) and 0.3 mL of 10% aluminum chloride (AlCl_3_). After 5 min, 2 mL of 1 M sodium hydroxide (NaOH) was added, and the volume was made up to 10 mL using distilled water. After thorough mixing, the absorbance was measured at 510 nm using a spectrophotometer (SH-U830, Shishin Technologies Co., Ltd., Taipei City, Taiwan), and the TFC (mg QE/g) of the sample was determined using a standard curve prepared with Quercetin (Sigma-Aldrich, St. Louis, MO, USA) at the concentrations ranging from 0.004 to 0.06 mg quercetin/mL (Figure S1C). In contrast, the result was performed in mg quercetin/g to determine the total flavonoid content (mg quercetin/g DW) of the samples.

#### 2.2.4. Chlorophyll

Freeze-dried sample powder (0.1 g) was mixed with 1 mL of dimethyl sulfoxide (DMSO) and heated in a 65 °C water bath for 15 min. The mixture was centrifuged (model: Heraeus Megafuge 8R, ThermoFisher, Onlyscience Co., Ltd., Taichung City, Taiwan) at 2268× *g* for 10 min and the supernatant was collected. The precipitate was re-extracted by adding 1 mL of DMSO, and the supernatants collected after centrifugation were combined. Chlorophyll content (mg/g) was determined using a spectrophotometer at wavelengths of 645 nm and 663 nm using a spectrophotometer (model: SH-U830, Shishin Technologies Co., Ltd., Taipei City, Taiwan) and calculated using the following Formulas (1) and (2).

Chlorophyll a = [(12.7 × A 663) − (2.69 × A 645)] × mL DMSO/mg djulis leaves(1)

Chlorophyll b = [(22.9 × A 645) − (4.68 × A 663)] × mL DMSO/mg djulis leaves(2)

At wavelengths 645 nm and 663 nm, the absorbance is represented by A 645 and A 663, respectively.

#### 2.2.5. Betacyanin and Betaxanthin

The analysis of betacyanin (BC) and betaxanthin (BX) in djulis leaf was conducted using high-performance liquid chromatography (HPLC) [6]. Djulis samples were first freeze-dried and finely ground into a uniform powder. The pigments were extracted by mixing 1 g of djulis powder with 20 mL of 80% methanol containing 0.1% formic acid, followed by ultrasonication for 30 min at 25 °C. The extract was centrifuged at 10,000 rpm for 15 min, and the supernatant was filtered through a 0.45 µm membrane filter before analysis.

The HPLC analysis was performed using a reverse-phase C18 column (4.6 × 250 mm, 5 µm particle size). The mobile phase consisted of solvent A (0.1% formic acid in water) and solvent B (0.1% formic acid in acetonitrile) with a gradient elution program: 0–5 min, 95% A; 5–15 min, 95–75% A; 15–20 min, 75% A. The flow rate was set at 1.0 mL/min, and the injection volume was 20 µL. Detection was carried out using a diode array detector (DAD) at 538 nm for betacyanin and 476 nm for betaxanthin. The identification and quantification of the pigments were achieved by comparing the retention times and absorption spectra with those of standard betacyanin and betaxanthin compounds. A standard curve for betacyanin was prepared using concentrations ranging from 1.0 to 6.0 μg/g, while the standard curve for betaxanthin used concentrations ranging from 1.0 to 8.0 μg/g.

### 2.3. Antioxidant Capacity

#### 2.3.1. DPPH Free Radical Scavenging Assay

Freeze-dried sample powder (1 mg) was dissolved in methanol to prepare the sample solution. Then, 0.1 mL of the sample solution was mixed with 0.9 mL of DPPH (2,2-diphenyl-1-picrylhydrazyl) solution (0.15 mM in methanol) [17]. The reaction mixture was incubated at room temperature in the dark for 30 min. The absorbance was measured at 517 nm using a spectrophotometer.

#### 2.3.2. Ferric-Reducing Antioxidant Power (FRAP)

FRAP solution was prepared by mixing 0.1 M acetate buffer (pH 3.6), 10 mM ferric-tripyridyltriazine (TPTZ, 0.01 M), and 20 mM ferric acid (0.02 M) at a ratio of 10:1:1 (*v/v/v*) [17]. Freeze-dried sample powder (1 mg) was dissolved in distilled water to prepare the sample solution. Then, 0.1 mL of the sample solution was mixed with 1.9 mL of FRAP solution (Figure S2A). After incubation at room temperature for 10 min, absorbance was measured at 593 nm using a spectrophotometer (SH-U830, Shishin Technologies Co., Ltd., Taipei City, Taiwan).

#### 2.3.3. Trolox Equivalent Antioxidant Capacity (TEAC)

The 2,2′-azino-bis-(3-ethylbenzothiazoline-6-sulfonic acid) (ABTS^•+^) solution was prepared by dissolving 20 mg of ABTS in 2.6 mL of 0.0049 mol/L potassium persulfate (K_2_S_2_O_8_) and 2.6 mL of distilled water [17]. Absorbance was measured at 734 nm. The ABTS solution was diluted with phosphate buffer (5 mmol/L, pH 7.4) to achieve an absorbance of 0.700 ± 0.020 (Figure S2B). The freeze-dried sample powder was dissolved in distilled water, and 10 μL of the sample solution was mixed with 900 μL ABTS solution. The absorbance was measured at 734 nm using a spectrophotometer (SH-U830, Shishin Technologies Co., Ltd., Taipei City, Taiwan).

#### 2.3.4. N, N-Dimethyl-p-Phenylenediamine (DMPD)

A 100 mM DMPD solution was prepared by dissolving 209 mg of DMPD in 10 mL of distilled water [17]. The DMPD solution (1 mL) was mixed with 100 mL of 0.1 M acetate buffer (pH 5.3). The freeze-dried powder samples were dissolved in distilled water, and 200 μL of the sample solution was mixed with 2 mL of DMPD solution. After incubation at room temperature in the dark for 15 min, absorbance was measured at 505 nm using a spectrophotometer (SH-U830, Shishin Technologies Co., Ltd., Taipei City, Taiwan).

#### 2.3.5. Cupric-Ion-Reducing Antioxidant Capacity Assay (CUPRAC)

A 10 mM copper (II) chloride solution, 7.5 mM neocuproine solution, and 1 M ammonium acetate buffer (pH 7.0) solution were prepared [17]. The freeze-dried powder samples were dissolved in distilled water. Then, 100 μL of the sample solution was mixed with 1 mL of copper (II) chloride solution, neocuproine solution, ammonium acetate buffer (pH 7.0) solution, and distilled water. After incubation at room temperature for 30 min, absorbance was measured at 450 nm using a spectrophotometer (SH-U830, Shishin Technologies Co., Ltd., Taipei City, Taiwan) (Figure S2C).

### 2.4. Dermatology Skin Test

Table 1 shows the compositions of the djulis leaf extract test formulations, which were prepared based on the previous method with modifications [21]. The carbopol gel was prepared using hot water. Next, djulis leaf extract, glycerin, and triethanolamine (TEA) were added and stirred vigorously. The gel sample without djulis leaf extract was designated as the control (blank, B) sample. Gel samples containing 0.0625% (*w/v*) of green, red, or yellow djulis leaf extract were labelled as G-1, R-1, and Y-1, respectively. Gel samples containing 0.125% (*w/v*) of green, red, or yellow djulis leaf extract were labelled as G-2, R-2, and Y-2, respectively. Gel samples containing 0.25% (*w/v*) of green, red, or yellow djulis leaf extract were labelled as G-3, R-3, and Y-3, respectively.

The color of the formulated djulis leaf gel samples was evaluated using a Color Meter ZE-2000 (Nippon Denshku Industries Co., Ltd., Tokyo, Japan). The parameters L* (lightness), a* (green-to-red spectrum, where negative values indicate a shift towards green and positive values indicate a shift towards red), and b* (blue-to-yellow spectrum, where negative values indicate a shift towards blue and positive values indicate a shift towards yellow) were recorded to assess variations in color quality. Before measurement, the device was calibrated with a standard black-and-white ceramic tile. All color assessments were conducted at room temperature, with each sample measured in triplicate.

The test samples were freshly prepared prior to testing. Approximately 35 students from the Department of Food and Nutrition, Providence University (Taichung City, Taiwan), were interviewed, and 30 were selected to participate in the single-application closed-patch epicutaneous test under semi-occlusion conditions. Before participating in the study, the participants were required to provide written informed consent. The participants completed the personal data form. Skin properties such as the moisture, oil, texture, complexion, and 3D levels were analyzed using a skin analyzer (deViso skin analyzer, Prismatique, FL, USA) at the beginning of the test. Next, 1 mL of the test sample was dipped into the cotton pad and placed on the inner wrist. The control sample contained sterilized water on the wrist instead of djulis leaf extracts. After 20 min, the skin properties were analyzed using a skin analyzer (Multi Skin Test Center MC 1000, Courage-Khazaka Electronic GmbH, Cologne, Germany).

This study protocol followed Good Clinical Practices and the Declaration of Helsinki and agreed with the appropriate institutional review board (IRB) regulations. The experimental protocol was registered at the Taichung Jen Ai Hospital. This protocol was approved by the IRB of Taichung Jen Ai Hospital, Taichung, Taiwan (clinical trial approval certificate no. 110-59). The participants gave informed consent before beginning any study-related procedures or medications.

### 2.5. Statistical Analysis

All analyses of physicochemical parameters and antioxidant capacities were conducted in triplicate (n = 3), and the data are expressed as mean values ± standard deviations (X ± SD). Statistical analyses were carried out using Microsoft Office 365 for data calculations, with one-way ANOVA performed and Duncan’s multiple range test applied to assess significant differences at *p* < 0.05 using SPSS software (version 23.0; SPSS Inc., Chicago, IL, USA). Additionally, correlation analysis was performed using XLSTAT software (version 2020; Addinsoft, New York, NY, USA).

## 3. Results and Discussion

### 3.1. Physicochemical Properties of Djulis Leaves

**Figure 2:**
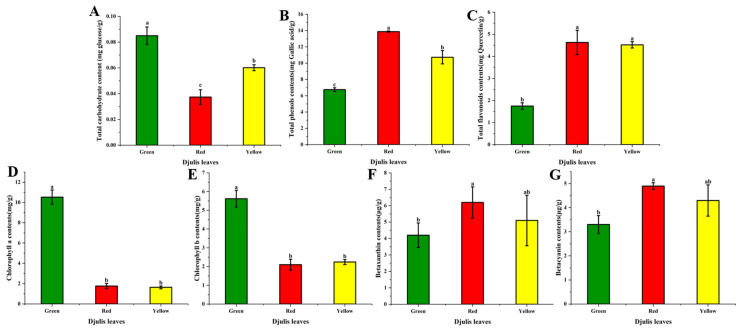
Bioactive compound content of djulis leaves at different senescence stages. (**A**) Total carbohydrate content (mg glucose/g DW), where ‘mg/g DW’ represents milligrams of carbohydrate per gram of dry weight. (**B**) Total phenolic content (mg gallic acid/g DW), quantified as milligrams of gallic acid equivalents per gram of dry weight. (**C**) Total flavonoid content (mg quercetin/g DW), expressed as milligrams of quercetin equivalents per gram of dry weight. (**D**) Chlorophyll a content (mg/g DW). (**E**) Chlorophyll b content (mg/g DW). (**F**) Betaxanthin content (μg/g DW). (**G**) Betacyanin content (μg/g DW). The results highlight significant differences in the bioactive profiles of green, red, and yellow djulis leaves. Error bars represent standard deviations (n = 3). Means with different letters indicate statistically significant differences at *p* < 0.05 based on Duncan’s multiple range test.

The amount of carbohydrates present in the djulis leaf extracts was determined using the phenol-sulfuric acid method. This method involves the hydrolysis of carbohydrates and subsequent reaction with concentrated sulfuric acid, resulting in a yellow coloration that is directly proportional to the carbohydrate content. The total carbohydrate content of the three djulis leaf extracts was assessed, as shown in Figure 2A. Among the djulis leaf extracts, the highest total carbohydrate content was observed in green djulis leaves (0.09 mg/g), followed by yellow (0.06 mg/g) and red djulis leaves (0.04 mg/g).

Phenolic compounds are prevalent functional substances in plants, recognized for their antioxidative properties and health benefits, including their role in reducing stress-induced ailments [22]. The total phenolic content was determined using a reduction assay, in which phenolic compounds in the sample were oxidized to form dark-colored compounds, with absorbance directly proportional to TPC. Figure 2B illustrates the TPC of djulis leaf extracts, with red djulis leaves exhibiting the highest content (13.88 mg GA/g), followed by yellow (10.73 mg GA/g), and green djulis leaves had the lowest (6.75 mg GA/g). Variations in phenolic content among djulis leaf extracts may arise from differences in pigment synthesis pathways or external environmental factors such as light exposure, temperature, moisture, and nutrients during growth [17].

Flavonoids, another subclass of polyphenolic compounds widely present in plants, exhibit numerous health benefits including anti-inflammatory and antioxidant properties [6]. The TFC of djulis leaf extracts was determined, and the results are presented in Figure 2C. Among the extracts, red djulis leaves showed the highest flavonoid content (4.46 mg QE/g), followed by yellow (4.52 mg QE/g), and green djulis leaves had the lowest content (1.75 mg QE/g). The correlation between flavonoid and phenolic content suggests similar biosynthetic pathways or regulatory mechanisms governing their accumulation [23].

Chlorophylls serve as indicators of plant coloration and are vital pigments in photosynthesis, aiding in the conversion of carbon dioxide to organic compounds and oxygen release [24]. The chlorophyll content (a and b) of the djulis leaf extracts was assessed and is presented in Figure 2D,E. Green djulis leaves exhibited the highest chlorophyll content (10.53 mg/g; 5.62 mg/g), characterized by deep-green coloration. Conversely, yellow and red djulis leaves displayed lower chlorophyll content, corresponding to their respective coloration. The chlorophyll content in the current study was higher than the previous study that investigated the effect of calcium on the antioxidant enzyme activity of djulis sprouts [25].

Betacyanins and betaxanthins are natural pigments commonly found in plants, imparting purple–red and orange–yellow hues, respectively [26]. The betaxanthin (BX) and betacyanin (BC) content of djulis leaf extracts was determined and is presented in Figure 2F,G (Figure S3). The green djulis leaf extract exhibited the lowest content of both BX and BC (4.2 μg/g and 3.3 μg/g, respectively), while yellow and red djulis leaf extracts showed slightly higher levels.

The diverse phytochemical profiles observed in red, green, and yellow djulis leaf extracts offer valuable insights into the dynamic biochemical changes that occur during leaf senescence. The variation in total carbohydrate content among the extracts reflects the shifting metabolic priorities and energy storage strategies across different stages of leaf development [27]. Green djulis leaves, representing an early senescence stage, exhibited the highest total carbohydrate content, which is indicative of active photosynthesis and carbohydrate accumulation to support growth and development. In contrast, the lower total carbohydrate content observed in red djulis leaves, representing a later senescence stage, suggests a shift toward carbohydrate utilization or allocation to other metabolic pathways [28]. Additionally, seasonal and environmental factors, such as temperature, soil nutrient availability, and water stress, may influence the antioxidant profiles of djulis leaves. For instance, higher temperatures or water scarcity during specific growing seasons can induce oxidative stress in plants, leading to the increased production of secondary metabolites like phenolics and flavonoids as part of the plant’s defense mechanism [29]. Similarly, variations in soil nutrient composition may impact the biosynthesis of pigments like betaxanthins and betacyanins, contributing to the differences observed in the phytochemical and antioxidant profiles of leaves harvested under different conditions. Future studies should consider these environmental variables to better understand the factors influencing the bioactive composition of djulis leaves.

Significant differences in TPC among the three djulis leaf extracts highlight their varying antioxidant capacities. Red djulis leaves, characterized by advanced senescence, displayed the highest TPC, potentially as a response to increased oxidative stress and environmental challenges. Conversely, green djulis leaves exhibited relatively low TPC, reflecting their earlier developmental stage and reduced demand for antioxidant defense. These findings highlight the dynamic modulation of phenolic metabolism during leaf senescence and its implications for plant physiology and stress response [30].

Furthermore, the variation in TFC among the extracts underscores the dynamic regulation of secondary metabolites during leaf development. The higher flavonoid content observed in red and yellow djulis leaves may serve as a protective mechanism against environmental stressors and pathogens, particularly during the later stages of senescence when plants are more vulnerable [31]. In contrast, green djulis leaves showed relatively low flavonoid content, reflecting their early developmental stage and potentially reduced need for flavonoid-mediated defense mechanisms.

Chlorophyll content analysis revealed distinct pigment composition patterns across the different senescence stages of djulis leaves. Green djulis leaves exhibited the highest chlorophyll content, consistent with their active photosynthetic machinery and primary role in light absorption and energy capture [32]. As leaves mature, their chlorophyll content decreases, causing changes in pigment composition and color [32]. The observed variations in chlorophyll a and chlorophyll b content among djulis leaf extracts highlight the dynamic nature of chlorophyll metabolism during leaf development and its impact on photosynthetic efficiency and pigment composition.

The determination of betaxanthin and betacyanin content provides insights into the pigment profile of djulis leaves at different senescence stages. The higher betaxanthin and betacyanin content observed in red djulis leaves may be attributed to enhanced pigment accumulation during senescence stages, contributing to their distinctive red coloration [6]. Conversely, green djulis leaves displayed relatively lower betaxanthin and betacyanin content, consistent with their early developmental stage and reduced pigment synthesis. These findings underscore the dynamic regulation of pigment biosynthesis pathways during leaf senescence and their role in determining leaf coloration and visual appeal.

### 3.2. Antioxidant Capacity of Djulis Leaves

**Figure 3:**
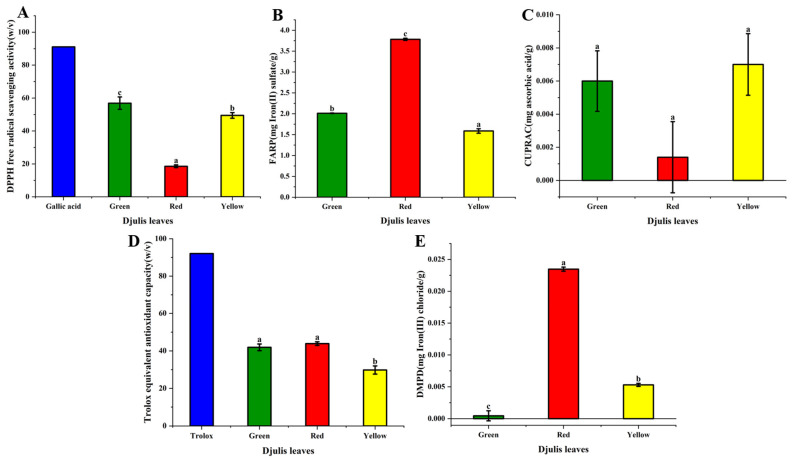
Antioxidant activities of djulis leaf extracts at different senescence stages measured by various in vitro assays: (**A**) DPPH radical scavenging activity (%, *w/v*), expressed as the percentage of free radical scavenging relative to the standard (gallic acid). (**B**) Ferric-reducing antioxidant power (FRAP), expressed in mg FeSO₄ equivalents per gram of dry weight (mg FeSO₄/g DW). (**C**) Cupric-reducing antioxidant power (CUPRAC), expressed in mg ascorbic acid equivalents per gram of dry weight (mg ascorbic acid/g DW). (**D**) Trolox equivalent antioxidant capacity (TEAC), expressed as a percentage of antioxidant capacity compared to the Trolox standard. (**E**) DMPD antioxidant capacity, expressed in mg ferric chloride equivalents per gram of dry weight (mg FeCl₃/g DW). These units provide a standardized measure of antioxidant activity, enabling comparisons across different assays and extracts. Error bars represent standard deviations (n = 3). Means with different letters indicate statistically significant differences at *p* < 0.05 based on Duncan’s multiple range test.

DPPH (2, 2-diphenyl-1-picrylhydrazyl) is commonly used to assess the antioxidant capacity of free radicals. The antioxidant ability of the samples was evaluated by measuring their ability to scavenge DPPH radicals. Antioxidants in the sample donate electrons to DPPH radicals, reducing them. This reduction process changes the deep purple color of the DPPH radicals to a light yellow or colorless hue, accompanied by a decrease in absorbance [17]. In this experiment, the DPPH free radical scavenging ability of the three djulis extracts was determined, as shown in Figure 3A, with gallic acid used as a control. Green djulis leaves exhibited the highest DPPH radical scavenging ability (56.91%, *w/v*), followed by yellow djulis leaves (49.45%, *w/v*), while red djulis leaves displayed the lowest activity (18.58%, *w/v*).

The FRAP assay is a simple, rapid, and reproducible method for assessing the antioxidant capacity. It evaluates the ability of the sample to reduce ferric ions (Fe^3+^) to ferrous ions (Fe^2+^), reflecting its antioxidant potential [33]. The FRAP values of the three extracts were determined, as shown in Figure 3B. The red djulis leaves had the highest FRAP value (3.78 mg FeSO_4_/g), followed by the green djulis leaves and yellow djulis leaves, with values of 2.01 mg FeSO_4_/g and 1.59 mg FeSO_4_/g, respectively.

The CUPRAC assay is based on the reduction in copper ions (Cu^2+^) to copper ions (Cu^+^), primarily used to assess the sample’s reducing power. In this experiment, the copper ion reduction ability of three djulis extracts was determined, as shown in Figure 3C. The CUPRAC values of green, yellow, and red djulis leaf extracts were 0.006 mg ascorbic acid/g, 0.007 mg ascorbic acid/g, and 0.0014 mg ascorbic acid/g, respectively, while yellow djulis leaves exhibited the highest activity, followed by green djulis leaves, and the lowest was shown by red djulis leaves.

The Trolox equivalent antioxidant capacity (TEAC) is an analytical method used to measure the antioxidant capacity of the samples. It assesses the ability of the sample to scavenge a synthetic antioxidant (Trolox), and reflects its overall antioxidant potential. Using 2,2’-Azino-bis(3-ethylbenzothiazoline-6-sulfonic acid) diammonium salt (ABTS) reagent, the assay generates ABTS^•+^ radicals, and the ability of the sample to quench ABTS^•+^ radicals was measured [33]. In this experiment, the TEAC of three djulis leaf extracts was determined, and Figure 3D illustrates the results, with Trolox serving as a control sample (92.1%, *w/v*). Red djulis leaves had the highest antioxidant capacity (43.9%, *w/v*), followed by green djulis leaves (41.9%, *w/v*), while yellow djulis leaves exhibited the lowest antioxidant capacity (29.8%, *w/v*).

The DMPD assay was used to determine the antioxidant capacity of the samples based on the electron transfer reaction between the reagent and the antioxidants in the sample. This assay assesses the ability of the samples to transfer electrons, changing the color of the DMPD radical cation from blue to colorless or pale. In this experiment, the DMPD antioxidant capacity of the three djulis extracts was determined, as shown in Figure 3E; the red djulis leaves exhibited the highest DMPD value (0.024 mg FeCl_3_/g), followed by the yellow djulis leaves (0.005 mg FeCl_3_/g), while the green djulis leaves had the lowest value of only 0.0005 mg FeCl_3_/g. Compared to other natural ingredients commonly used in cosmeceutical products, such as green tea, which is rich in catechins—particularly epigallocatechin gallate (EGCG), known for its strong radical-scavenging ability as demonstrated by DPPH and FRAP assays—red djulis leaf extract shows noteworthy phenolic content and antioxidant capacity [34]. This suggests that it may play a competitive or complementary role in formulations. Furthermore, the betaxanthin and betacyanin content of red djulis leaves provides additional antioxidative benefits, distinguishing it from these conventional ingredients. These unique properties position djulis leaves as a promising and sustainable ingredient for cosmeceutical development, with the potential to cater to growing demands for natural and eco-friendly products.

The observed variations in antioxidant activities among red, green, and yellow djulis leaf extracts provided valuable insights into the dynamic antioxidant capacity at different senescence stages. Antioxidant assays, including DPPH, FRAP, CUPRAC, TEAC, and DMPD, reflect the diverse phytochemical profiles and oxidative stress responses exhibited by djulis leaves during senescence. The DPPH assay, commonly used to assess free radical scavenging activity, revealed significant differences in antioxidant capacity among red, green, and yellow djulis leaf extracts. The highest DPPH scavenging activity was observed in the green djulis leaf extract, indicating its potent antioxidant properties. This may be attributed to the presence of high levels of chlorophyll compounds, which are known to contribute to antioxidant activity. Conversely, red and yellow djulis leaf extracts exhibited relatively lower DPPH scavenging activity, suggesting variations in antioxidant composition and efficacy across different senescence stages [35].

Similarly, the FRAP and CUPRAC assays, which evaluate the ferric-reducing antioxidant power, provided insights into the reducing capacity of red, green, and yellow djulis leaf extracts. The red djulis leaf extract displayed the highest FRAP values, whereas yellow djulis leaves exhibited the highest CUPRAC value, indicating superior ferric-reducing ability. In contrast, yellow and red djulis leaf extracts exhibited lower FRAP and CUPRAC values, suggesting differences in their capacity to donate electrons and reduce oxidized species. These findings underscore the influence of senescence stage on antioxidant activity and the potential implications for oxidative stress mitigation and cellular protection [35].

Moreover, the TEAC assay, which measures the ability of antioxidants to scavenge ABTS radicals, revealed distinct antioxidant capacities among red, green, and yellow djulis leaf extracts. While all extracts exhibited TEAC values indicative of antioxidant activity, the red djulis leaf extract demonstrated the highest TEAC value, followed by the green and yellow djulis leaf extracts. This variation in TEAC values may be attributed to differences in the phytochemical composition, particularly flavonoids and phenolic acids, which contribute to the scavenging of ABTS radicals and other reactive oxygen species [33].

Additionally, the DMPD assay evaluates the antioxidant capacity based on electron transfer reactions, providing further insights into the antioxidant properties of the three djulis leaf extracts. The red djulis leaf extract exhibited the highest DMPD value, indicating its superior electron transfer ability and potential for oxidative stress mitigation. Conversely, yellow and green djulis leaf extracts displayed lower DMPD values, suggesting differences in their capacity to donate electrons and neutralize free radicals. The comprehensive evaluation of antioxidant activities in djulis leaf extracts highlights the dynamic antioxidant capacity at different senescence stages. The observed variations in DPPH, FRAP, CUPRAC, TEAC, and DMPD values underscore the complex interplay between the phytochemical composition, senescence stage, and antioxidant efficacy.

### 3.3. Color and Appearance of Djulis Leaf Gel

**Figure 4:**
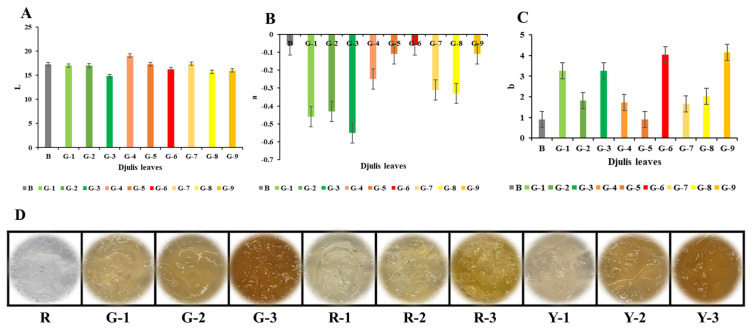
(**A**) L*, (**B**) a*, and (**C**) b* values representing the color parameters of djulis leaf gels, and (**D**) visual appearance of the gels prepared from different djulis leaf extracts. Abbreviations: B refers to the blank control gel (no djulis extract added); G-1, G-2, and G-3 refer to gels prepared from green djulis leaf extracts at three different concentrations; R-1, R-2, and R-3 refer to gels prepared from red djulis leaf extracts at three different concentrations; Y-1, Y-2, and Y-3 refer to gels prepared from yellow djulis leaf extracts at three different concentrations. Error bars represent standard deviations (n = 3).

The color changes in the gels with djulis extract are illustrated in Figure 4A–C. The gel matrix was transparent, but with the addition of djulis extract, the gel turned yellow. As the concentration of the djulis extract increased (from 0.0625% to 0.25%), color changes were observed. The L^*^ values of the gels with red, green, and yellow djulis leaves decreased, and higher concentrations of the djulis extract resulted in gels that were yellow and opaque. Variations in the djulis leaf extracts led to different gel colors. The addition of red djulis leaf extract resulted in gels with colors ranging from tan to dark brown as the concentration increased. The gel color changed from light yellow to golden yellow with increasing concentrations of green djulis leaves. Meanwhile, the addition of yellow djulis leaves results in a color change from yellow to deep yellow. A higher a^*^ value indicates a more yellow color and, in gels containing 0.25% djulis extract, the a^*^ values for red, green, and yellow djulis leaves (−0.11 ± 0.25, −0.55 ± 0.15, and −0.11 ± 0.09, respectively) showed higher values for red and yellow djulis leaf gels. In contrast, a higher b^*^ value indicates a more yellow color. In gels containing red, green, and yellow djulis extracts (−0.11 ± 0.25, −0.55 ± 0.15, and −0.11 ± 0.09, respectively), the b^*^ values are higher for red and yellow djulis extract gels, which is consistent with the leaf color results.

The observed variations in the color of gels supplemented with djulis leaf extract can be attributed to multiple factors. First, the inherent differences in pigment composition and chemical properties of the djulis leaf extracts contributed significantly to the color variations observed. The presence of different pigments in djulis leaves results in varying color responses when used in gel matrices [36]. Additionally, the antioxidant activity of these pigments may influence gel formation mechanisms and color stability during gelation processes [37]. Furthermore, the gel color changes observed, such as the transition from transparent to yellow hues with increasing concentrations of djulis extract, align with the expected changes in L^*^, a^*^, and b^*^ values indicative of color shifts towards higher yellowness. These findings underscore the intricate interplay between pigment composition, antioxidant activity, gel formation dynamics, and resultant color changes, providing valuable insights into the development of functional food products enriched with djulis extracts.

### 3.4. Dermatological Test of Djulis Leaf Gel

**Figure 5:**
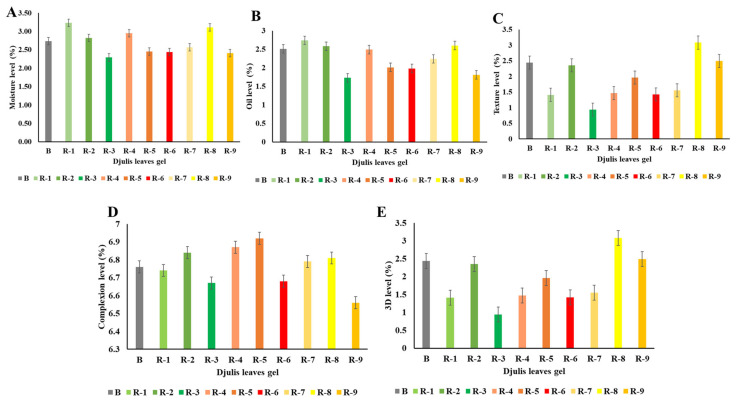
Dermatological analysis of djulis leaf gel formulations over 20 min, showing changes in (**A**) moisture level, (**B**) oil level, (**C**) texture level, (**D**) complexion level, and (**E**) 3D level. Abbreviations: B refers to the blank control gel (no djulis extract added); G-1, G-2, and G-3 represent gels prepared from green djulis leaf extracts at three different concentrations; R-1, R-2, and R-3 represent gels prepared from red djulis leaf extracts at three different concentrations; Y-1, Y-2, and Y-3 represent gels prepared from yellow djulis leaf extracts at three different concentrations. Error bars represent standard deviations (n = 3).

The experiment aimed to evaluate skin properties after applying gel formulations containing djulis leaf extracts for 20 min. The results depicted in Figure 5 illustrate the changes in the skin characteristics. Figure 5A (moisture level) and E (3D level) indicate an upward trend in moisture and 3D level, while Figure 5B (oil level), C (texture level), and D (complexion level) display a downward trend in oil secretion, skin texture, and complexion, respectively. Upon the application of djulis leaf extract gels for 20 min, the gel containing yellow djulis leaf extract decreased skin oiliness, texture irregularities, and discoloration, thereby improving skin wrinkles, inhibiting oil secretion, and reducing skin dullness. Red djulis leaf gel led to an increase in skin 3D level, while green djulis leaf gel enhanced skin hydration, augmenting its moisture content. After applying the djulis leaf extract gel formulations to the inner forearm, testers noticed significant improvements in skin hydration, elasticity, radiance, and firmness, and reduced wrinkle scores. These positive effects on skin quality and functionality suggest the potential application of beauty gel formulations containing djulis leaf extracts, highlighting their biocompatibility and biodegradability with the skin.

The observed improvements in skin properties following the application of gel formulations containing djulis leaf extracts underscore the potential of these formulations in skincare applications. The significant increase in moisture levels and 3D skin texture, coupled with the reduction in oil secretion, texture irregularities, and complexion dullness, highlights the multifaceted benefits of djulis leaf extract on skin health. The distinct effects observed with different colored djulis leaf extracts suggest the presence of unique bioactive compounds in each extract, which may contribute to specific skin-enhancing properties [19]. The ability of yellow djulis leaf extract to mitigate oiliness and discoloration indicates its potential to address concerns related to acne-prone and uneven skin tones [38]. Conversely, the enhancement of 3D skin texture by red djulis leaf extract suggests its role in promoting skin firmness and elasticity [21]. Moreover, the hydration-improving effects of green djulis leaf extract emphasize its suitability for moisturizing skincare formulations [21]. These findings support the notion that djulis-leaf-extract-based gel formulations are promising natural and effective ingredients in skincare products, offering diverse benefits for improving skin health and appearance. Further research is necessary to fully understand the potential of these formulations for skincare, including their long-term effects and underlying mechanisms.

### 3.5. Correlation Analysis

**Figure 6:**
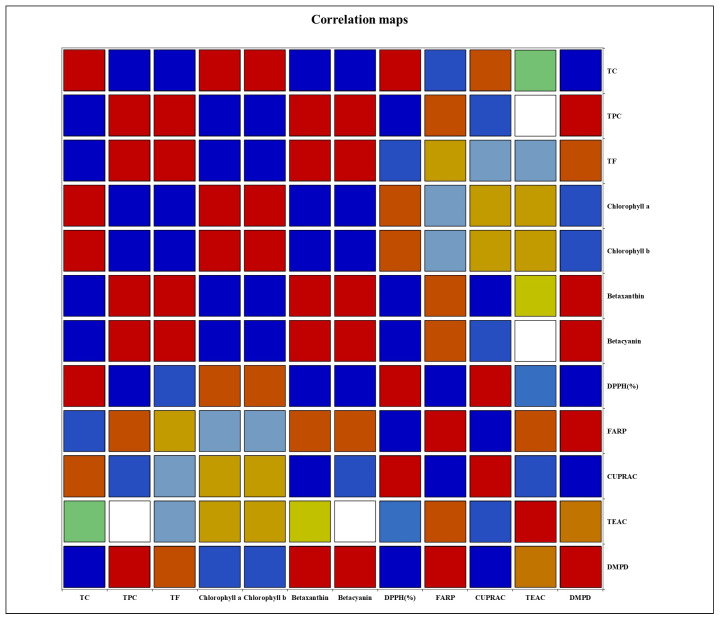
Correlation map showing the relationships between total carbohydrate (TC), total phenolic content (TPC), total flavonoid content (TF), chlorophyll a, chlorophyll b, betaxanthin, betacyanin, and antioxidant activities (DPPH, FRAP, CUPRAC, TEAC, and DMPD). Box colors represent the correlation coefficients: blue shades indicate negative correlations (darker blue = stronger negative correlation), red shades indicate positive correlations (darker red = stronger positive correlation), and white indicates no significant correlation.

Figure 6 and Figure S4 show the correlation analysis between the physicochemical properties and antioxidant capacity of three different colors of djulis leaves. The total carbohydrate content exhibited intriguing correlations, showing negative associations with TPC, TF, betaxanthin, betacyanin, and DMPD antioxidant activity, and was positively correlated with chlorophyll content and DPPH free radical scavenging activities. Conversely, TPC was negatively correlated with total carbohydrate, chlorophyll content, and DPPH antioxidant activity, while it was positively correlated with betaxanthin, betacyanin, and DMPD antioxidant activity. These correlations align with previous studies, which demonstrate the distinct roles of chlorophyll and phenolic compounds in antioxidant activity. Chlorophyll content has been positively correlated with DPPH free radical scavenging activity in other plants, as chlorophyll and its derivatives effectively quench reactive oxygen species (ROS) and reduce oxidative stress [29]. For example, Ferruzzi and Blakeslee (2007) highlighted the radical scavenging capacity of chlorophyll-derived compounds, such as pheophytins, which contribute to antioxidant activity in chlorophyll-rich plant extracts [39]. On the other hand, phenolic compounds, including TPC, have been shown to correlate with betacyanin and betaxanthin levels, as they share biosynthetic pathways under stress conditions that induce secondary metabolite production [40]. A related study also observed these associations in leafy vegetables, where chlorophyll levels and phenolic compounds exhibit inverse correlations due to different metabolic priorities during senescence [41]. Together, these comparisons emphasize that the antioxidant capacity of djulis leaves is influenced by the interplay of carbohydrate, chlorophyll, and phenolic compound levels across different senescence stages.

The significantly elevated DPPH free radical scavenging activities observed in green djulis leaf extracts align with increased total carbohydrate levels, leading to elevated chlorophyll a and chlorophyll b content. Additionally, the FRAP value was negatively correlated with total carbohydrates, whereas it was moderately positively correlated with TPC, betaxanthin, and betacyanin. Similarly, the DMPD assay exhibited negative correlations with total carbohydrates, but moderate positive correlations with TPC, betaxanthin, and betacyanin. These findings suggest that changes in TPC, betaxanthin, and betacyanin levels moderately influence the FRAP and DMPD activities of red djulis leaf extract. In contrast, the CUPRAC assay demonstrated negative correlations with TPC, betaxanthin, and betacyanin, but moderately positive correlations with total carbohydrate and chlorophyll content. This underscores the impact of total carbohydrate and chlorophyll content on CUPRAC activity, particularly in the extract of green djulis leaves. These findings highlight the complex interplay between physicochemical composition and antioxidant capacity in djulis leaves, highlighting their potential as valuable sources of natural antioxidants with diverse health-promoting properties.

## 4. Conclusions

The color variation observed in djulis leaves is attributed to several factors, including differences in chlorophyll content, betacyanin and betaxanthin accumulation, and overall plant metabolism during different growth stages. Chlorophyll, the green pigment responsible for photosynthesis, undergoes degradation as the leaves mature, leading to changes in leaf coloration. Simultaneously, the accumulation of betacyanins and betaxanthins, which are water-soluble pigments responsible for red hues in plants, contributes to the vibrant red color observed in mature djulis leaves.

The findings from this study elucidate the intricate relationship between the physicochemical composition and antioxidant capacity of red, yellow, and green djulis leaf extracts. The negative correlations observed between the total carbohydrate content and TPC, betaxanthin, and betacyanin underscore the dynamic balance between carbohydrate metabolism and the accumulation of antioxidant compounds. The notably high DPPH free radical scavenging activity observed in green djulis leaf extracts suggests a potential association with increased total carbohydrate levels, leading to elevated chlorophyll content. Moreover, the negative correlations of FRAP and DMPD assays with total carbohydrates, along with their positive correlations with TPC, betaxanthin, and betacyanin, highlight the influence of these antioxidant compounds on the overall antioxidant capacity of red djulis leaf extract. Conversely, the positive correlations between CUPRAC activities and total carbohydrate and chlorophyll content emphasize the impact of these components on the antioxidant potential, particularly in green djulis leaf extracts. In short, these findings enhance our understanding of the intricate relationship between the physicochemical composition and antioxidant capacity of djulis leaves. This suggests their potential as valuable sources of natural antioxidants for cosmeceutical products with diverse health-promoting properties. Red djulis leaves, rich in phenolic compounds and flavonoids, exhibit strong antioxidant activity, making them ideal for anti-aging creams aimed at reducing oxidative stress and preventing skin damage. Additionally, the betacyanin and betaxanthin content in red djulis leaves, known for their light-absorbing properties, could be utilized in UV protection lotions to enhance sun protection. Green djulis leaves, characterized by their high carbohydrate content, offer moisturizing and skin barrier repair properties, making them suitable for incorporation into hydrating gels or creams. These applications demonstrate the multifunctional potential of djulis extracts, providing a natural and sustainable alternative for cosmeceutical formulations.

While the current study provides valuable insights into the bioactive compounds and antioxidant efficacy of djulis leaves, several limitations should be acknowledged. First, the variability in bioactive compound content due to environmental factors such as climate, soil conditions, and cultivation practices can affect the reproducibility of results. Future studies should explore standardized agricultural practices to minimize such variability. Second, the scalability of the extraction process poses challenges, including potential cost and efficiency concerns. Optimizing extraction methods for industrial-scale production is critical for commercial applications. Finally, while the antioxidant potential of djulis leaves was demonstrated through in vitro assays, additional testing is required to evaluate their safety and efficacy in real-world cosmeceutical formulations. This includes cytotoxicity testing, skin irritation studies, and product stability assessments under various conditions. Addressing these limitations in future research will ensure the successful translation of djulis leaves into sustainable cosmeceutical products.

## Figures and Tables

**Table 1:** Composition of djulis gel.

Djulis Leaves Extracts (g)	Water (mL)	Carbopol (g)	TEA (g)	Glycerin (g)	Anti-Microbial Agent (g)
0	100	0.6	0.2	5	0.3
0.0625	100	0.6	0.2	5	0.3
0.125	100	0.6	0.2	5	0.3
0.25	100	0.6	0.2	5	0.3

## Data Availability

Data is contained within the article or Supplementary Materials.

## Supplementary Materials

The following supporting information can be downloaded at: https://www.mdpi.com/article/10.3390/antiox14020202/s1, Figure S1: (A) Standard curve of total carbohydrate content of glucose, (B) Standard curve of total phenols content of Gallic acid, and (C) Standard curve of total flavonoids content of quercetin of djulis leaves; Figure S2: (A) Standard curve of ferric reducing antioxidant power (FRAP) of ferrous sulfate, (B) Standard curve of cupric reducing antioxidant power (CUPRAC) of ascorbic acid, (C) Standard curve of trolox equivalent antioxidant capacity (TEAC) of trolox, and (D) Standard curve of DMPD antioxidant capacity of iron(III) chloride of djulis leaves; Figure S3: (A) Red djulis leaves, (B) Green djulis leaves, and (C) Yellow djulis leaves of HPLC analysis; Figure S4: Scatter plots of djulis leaves.
