# Supplementary material for: Bioactive Compounds and Antioxidant Efficacy of Djulis (Chenopodium formosanum) Leaves: Implications for Sustainable Cosmeceutical Development
Source: Antioxidants (Basel). 2025 Feb 10;14(2):202. doi: 10.3390/antiox14020202 (PMC11852302; doi:10.3390/antiox14020202)
Supplement: Supplementary file 1 [file antioxidants-14-00202-s001.zip › antioxidants-3397326-supp.pdf]

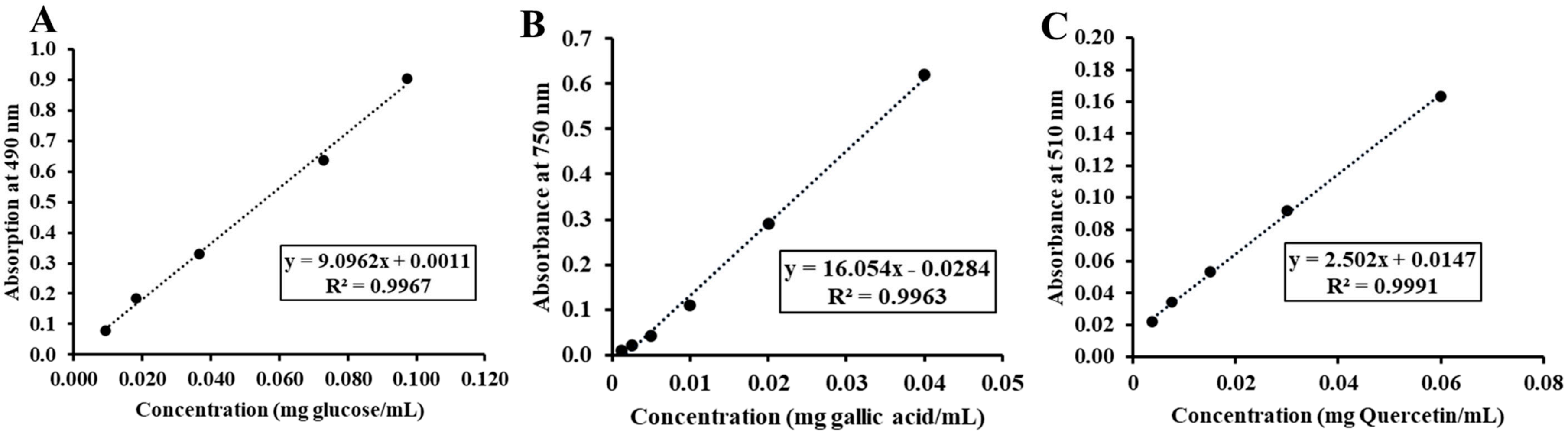

Figure S1: (A) Standard curve of total carbohydrate content of glucose, (B) Standard curve of total phenols content of Gallic acid, and (C) Standard curve of total flavonoids content of quercetin of djulis leaves.

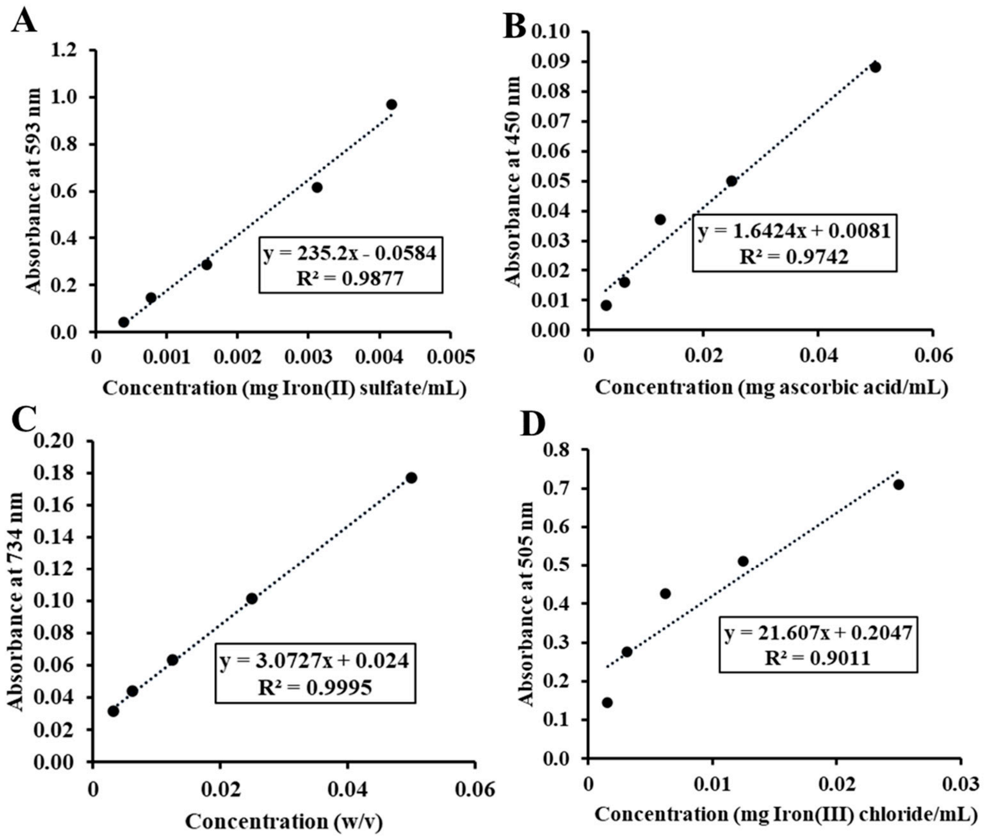

Figure S2: (A) Standard curve of ferric reducing antioxidant power (FRAP) of ferrous sulfate, (B) Standard curve of cupric reducing antioxidant power (CUPRAC) of ascorbic acid, (C) Standard curve of trolox equivalent antioxidant capacity (TEAC) of trolox, and (D) Standard curve of DMPD antioxidant capacity of iron(III) chloride of djulis leaves.

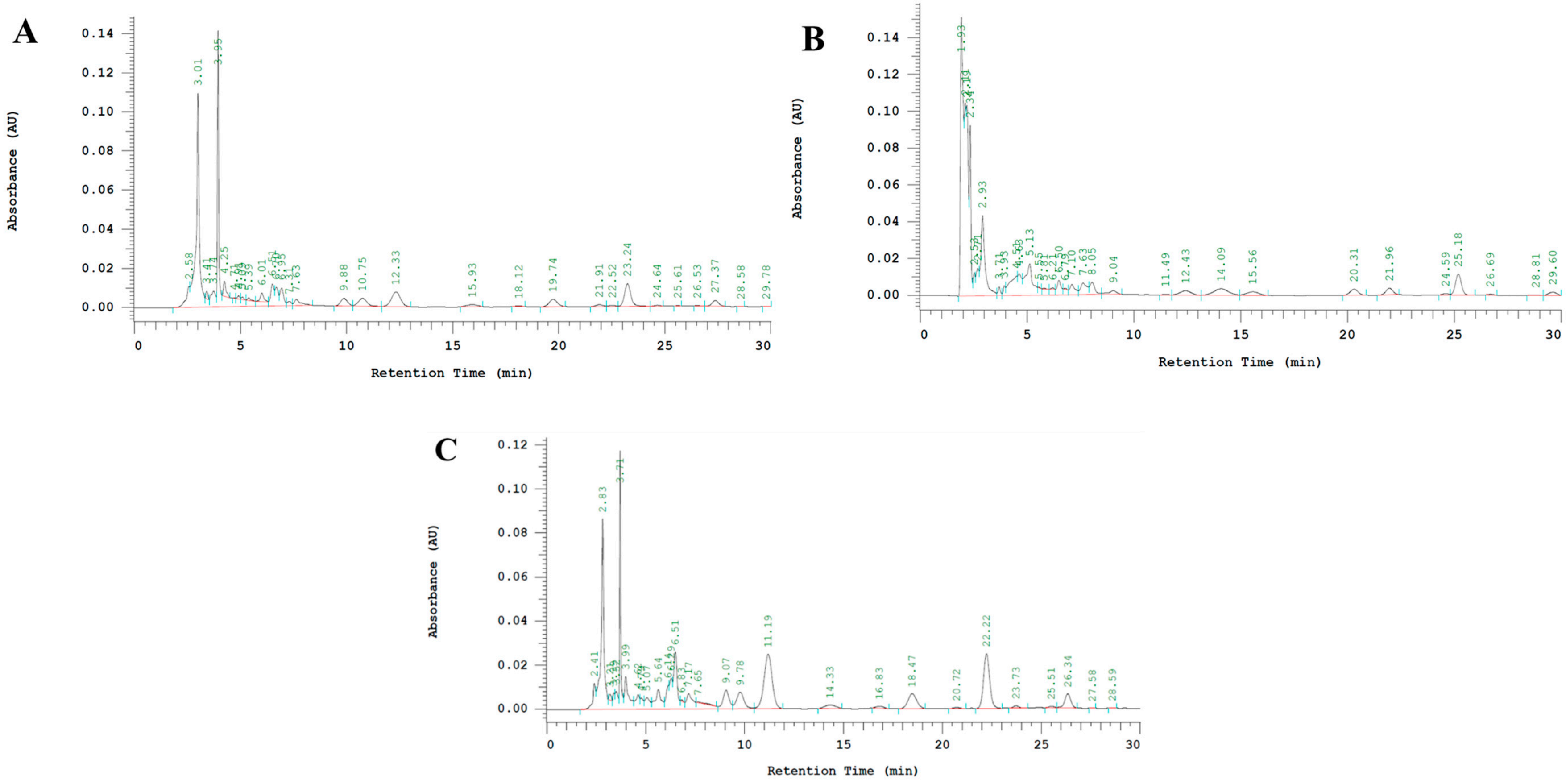

Figure S3: (A) Red djulis leaves, (B) Green djulis leaves, and (C) Yellow djulis leaves of HPLC analysis.

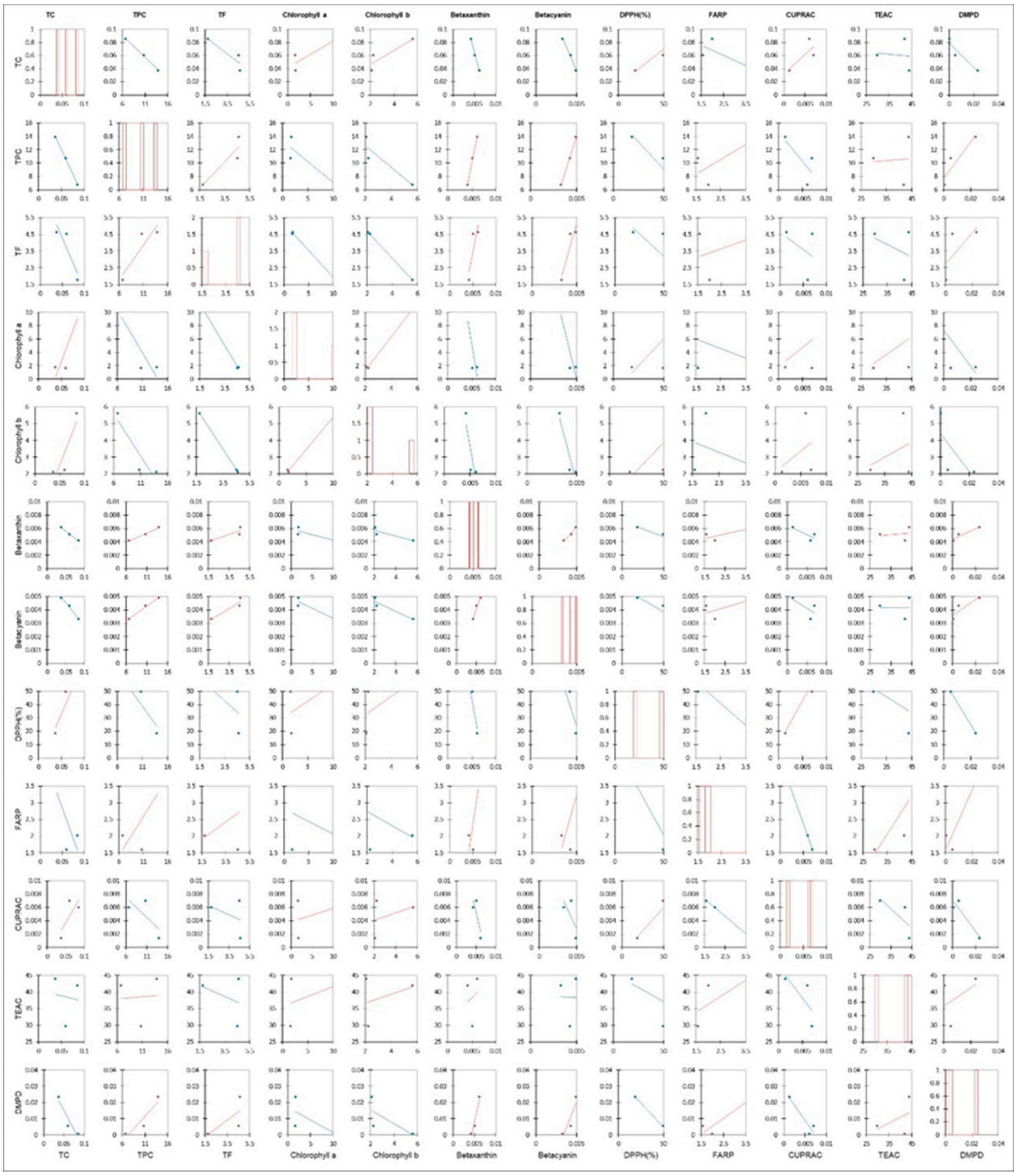

Figure S4: Scatter plots of djulis leaves.
